# Unleashing the power of antigen-presenting neutrophils

**DOI:** 10.1093/jmcb/mjae034

**Published:** 2024-09-16

**Authors:** Yingcheng Wu, Jiaqiang Ma, Qiang Gao

**Affiliations:** Department of Liver Surgery and Transplantation, and Key Laboratory of Carcinogenesis and Cancer Invasion (Ministry of Education), Liver Cancer Institute, Zhongshan Hospital, Fudan University, Shanghai 200032, China; Department of Liver Surgery and Transplantation, and Key Laboratory of Carcinogenesis and Cancer Invasion (Ministry of Education), Liver Cancer Institute, Zhongshan Hospital, Fudan University, Shanghai 200032, China; Department of Liver Surgery and Transplantation, and Key Laboratory of Carcinogenesis and Cancer Invasion (Ministry of Education), Liver Cancer Institute, Zhongshan Hospital, Fudan University, Shanghai 200032, China; Institutes of Biomedical Sciences, Fudan University, Shanghai 200032, China; State Key Laboratory of Genetic Engineering, Fudan University, Shanghai 200433, China

**Figure 1:**
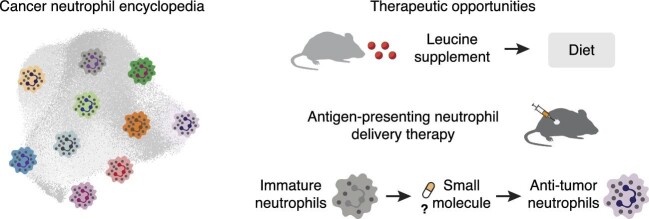
The cancer neutrophil encyclopedia: a roadmap for discovering novel neutrophil-based therapeutic strategies in anti-tumor treatment.

Neutrophils, the most abundant white blood cells in the human body, have long been recognized for their critical role in innate immunity (Quail et al., 2022). They serve as the first line of defense against invading pathogens and play a crucial role in inflammation and tissue repair (Hedrick and Malanchi, 2022). Our recent data have shed light on another facet of neutrophil biology: their potential as antigen-presenting cells (APCs) (Wu et al., 2024). By generating and integrating single-cell neutrophil transcriptomes from 17 cancer types, we observed 10 distinct neutrophil states, including an antigen-presenting state that was positively correlated with longer patient survival in multiple cancer types. We further demonstrated that leucine could upregulate expression levels of the major histocompatibility complex-II antigen-presenting molecule Human Leukocyte Antigen–DR (HLA-DR) and related co-stimulatory molecules in neutrophils, and these neutrophils could invoke both (neo)antigen-specific and antigen-independent T cell responses. Importantly, we showed that the delivery of leucine-activated antigen-presenting neutrophils or a leucine-rich diet could enhance the efficacy of anti-PD-1 therapy in various murine cancer models, highlighting the therapeutic potential of antigen-presenting neutrophils (Figure 1). Here, we will discuss the fascinating world of antigen-presenting neutrophils and outline the challenges and opportunities in harnessing their power for therapeutic purposes.

One of the challenges is developing more effective methods to reprogram neutrophils into APCs (Quail et al., 2022). While existing methods, using interferon-γ, granulocyte-macrophage colony-stimulating factor (GM-CSF), CD40L, and FcγRIIIB antibody–antigen conjugates, have shown promising effects (Oehler et al., 1998; Eruslanov et al., 2014; Singhal et al., 2016; Mysore et al., 2021), further optimization and exploration for novel approaches are still needed. GM-CSF has been shown to enhance the antigen-presenting capabilities of neutrophils, but its effects are transient and require continuous exposure. On the other hand, FcγRIIIB antibody–antigen conjugates have demonstrated the ability to target antigens specifically to neutrophils, but their efficacy in inducing a robust *in vivo* response remains to be fully characterized. Screening for small molecules or genetic modifications that can reprogram neutrophils into stable and potent APCs could lead to the development of more effective and targeted therapies.

Profiling the surfaceome of neutrophils is a critical step in designing effective neutrophil-targeted therapies, given the essential role of the cell surface in communication, adhesion, and targeting. By employing cutting-edge proteomic techniques, such as mass spectrometry and antibody-based arrays, future studies can comprehensively characterize the neutrophil surface proteome. This approach will enable the identification of novel neutrophil-specific markers that can be exploited for targeted drug delivery or the development of antibody-based therapies, providing valuable insights into the role of neutrophils in cancer pathogenesis. Additionally, investigating the dynamics of the neutrophil surface proteome during different stages of activation and differentiation could help identify key proteins involved in the acquisition of antigen-presenting functions. This knowledge could guide strategies to modulate neutrophil function and enhance their immunotherapeutic potential.

The spatial location of antigen-presenting neutrophils is another critical aspect in developing effective immunotherapies. While tumor-draining lymph nodes have been identified as key sites for neutrophil-mediated antigen presentation (Pylaeva et al., 2022), the role of neutrophils within other lymphoid organs, such as tertiary lymphoid structures (TLSs), remains unclear. TLSs are ectopic lymphoid organs that develop in response to chronic inflammation and are associated with improved prognosis in several types of cancer. However, it is still uncertain whether neutrophils reside within these structures or are merely transient visitors. Further research is needed to elucidate the dynamics of neutrophil trafficking and their interactions with other immune cells in TLSs. Understanding the spatial distribution and kinetics of antigen-presenting neutrophils within the tumor microenvironment could provide valuable insights for optimizing therapeutic strategies.

Finally, deciphering the interactions between antigen-presenting neutrophils and T cells is crucial for understanding antigen-presenting processes. The immunological synapse is a specialized junction formed between an APC and a T cell, facilitating the transfer of antigenic information and the activation of T cell responses (Basu and Huse, 2017). Elucidating the driving forces governing the formation and function of the neutrophil–T cell synapse could reveal new targets for intervention and help fine-tune the immune response. For instance, identifying the key receptors, adhesion molecules, and cytoskeletal components involved in the neutrophil–T cell synapse could lead to the development of small molecules or antibodies that can modulate the strength and duration of the synapse. Advances in imaging techniques, such as super-resolution microscopy as well as imaging flow cytometry, will be instrumental in unraveling the complexities of these interactions.

In summary, the therapeutic potential of harnessing antigen-presenting neutrophils remains vast yet untapped. While existing methods have shown promise in reprogramming neutrophils into APCs, further research is needed to develop effective strategies that reliably yield stable and potent APCs. The key may lie in identifying novel genetic or pharmacological targets that can flip the switch in neutrophils to transform them from short-lived cells into long-lived educators of the adaptive immune system. Advances in single-cell profiling and spatial mapping of immune cells and structures will elucidate when and where reprogrammed neutrophils need to be deployed for optimal efficacy. By deciphering the intricate molecular dance between neutrophils and T cells, we can learn to orchestrate their interactions to mount a robust anti-tumor immune response. With the continued unraveling of their biology, antigen-presenting neutrophils may prove to be powerful new allies in the fight against cancer. Their full potential awaits discovery by intrepid immunologists seeking new frontiers in immunotherapy.

*[We extend our sincere apologies for any omission of relevant citations and declare the use of an artificial intelligence language service to improve the writing. This study was supported by the National Natural Science Foundation of China (82130077, 81961128025, 82121002, and 82341008 to Q.G.; 823B2062 to Y.W.) and Shanghai Municipal Science and Technology Major Project. Q.G.: conceptualization, funding acquisition, providing resources, and supervision; Y.W. and Q.G.: writing the original draft; Y.W., J.M., and Q.G.: writing, review, and editing.]*
